# Protecting Men Who Have Sex With Men From HIV Infection With an mHealth App for Partner Notification: Observational Study

**DOI:** 10.2196/14457

**Published:** 2020-02-19

**Authors:** Xiangyu Yan, Zuhong Lu, Bo Zhang, Yongjie Li, Wenjun Tang, Lingling Zhang, Zhongwei Jia

**Affiliations:** 1 School of Public Health Peking University Beijing China; 2 National Institute on Drug Dependence Peking University Beijing China; 3 Biomedical Engineering Southeast University Jiangsu Province China; 4 College of Nursing and Health Sciences University of Massachusetts Boston Boston, MA United States

**Keywords:** partner notification, men who have sex with men, sexual network, mobile phone app

## Abstract

**Background:**

Traditional partner notification methods have been implemented for HIV-infected patients, as well as HIV treatment, in order to identify people at risk of HIV infection, especially men who have sex with men (MSM), since they are more likely to have casual sex partners. These traditional methods have some limitations.

**Objective:**

Our study focused on developing an mHealth app to improve partner notification in practice for MSM; the study then focused on evaluating the effects of the app.

**Methods:**

We developed an mHealth app with different modules using Java and HTML5 and tested it in an MSM community to prevent HIV transmission. The HIV incidence stratified by different follow-up periods were calculated. Poisson regression and social networks were used to estimate the risk ratios and to identify the connection among MSM, respectively.

**Results:**

In addition to the partner notification module, which is the kernel of the app, we developed a test result self-query module to enable MSM to get their approved test results in a timely manner, a prompt and warning module to alert users to protect themselves from high-risk conditions, and a health education module to teach users more skills regarding HIV/AIDS prevention. Over a 1-year duration, a total of 3186 MSM used the app, of which 678 had at least two HIV test results since becoming app users; they were included in the final analysis. Among 678 users, a total of 6473 self-queries and 623 partner notifications were recorded, which identified 180 social networks of MSM app users. Those who used the partner notification function were more likely to have self-queries (*P*<.001). The 678 MSM app users covered 296.47 person-years and contributed to 20 HIV seroconversions; the cumulative HIV infection incidence was estimated as 6.75 per 100 person-years (95% CI 4.38-10.01). We found that the longer the app was used, the lower the HIV incidence (>5 months vs ≤5 months: 2.22 per 100 person-years vs 6.99 per 100 person-years; risk ratio 0.32, 95% CI 0.12- 0.87).

**Conclusions:**

The app developed in this study is consistent with the World Health Organization’s sensitivity and confidentiality recommendations; it has the potential to reduce the risk of HIV infection among MSM.

## Introduction

HIV/AIDS is not only a serious infectious disease that endangers human health, but also a major global public health problem. In China, there were about 850,000 people reported to be living with HIV/AIDS in 2018; this number had increased rapidly over the years and is nearly double the number of about 440,000 people who were living with HIV/AIDS in 2011 [1,2]. Of the newly reported infections, 95.1% of people were infected through sexual transmission; 25.5% of them were men who have sex with men (MSM) and were infected though male-male sexual behaviors [3]. To control the spread of HIV effectively, providing antiretroviral therapy to people living with HIV/AIDS is a recommended method and is called *treatment as prevention* [4]. At present, antiretroviral therapy has covered about 75% of the reported HIV/AIDS cases worldwide and 80% of those in China, of which more than 90% were treated successfully [5,6]. However, as reported by the National Health Commission of China, about 30% of HIV-infected patients had not been found and did not know their HIV status [7]. As a result, they might not receive antiretroviral therapy and, therefore, this group comprises a tremendous number of latent sources of infection. As for MSM, the problem is particularly serious; because of the concealment of this subpopulation and stigma of HIV testing, it is difficult to find HIV-positive MSM in order to give them suitable treatment [8,9]. As most MSM seek sexual partners through Internet websites and software, such as Blued and Zank, including for one-night stands, they are more likely to hide their HIV status, which may increase the risk of HIV transmission [10,11]. Thus, it is important for them to know the risk of HIV infection and to know their real HIV status.

Partner notification is a public health measure that is recommended by the World Health Organization (WHO), in addition to *treatment as prevention*, to prevent and control HIV infection among HIV high-risk subpopulations, especially MSM [12-14]. Partner notification means that sexual partners should take the initiative to tell each other their HIV infection status; people living with HIV/AIDS, in particular, should tell their sexual partners their HIV status and encourage them to receive HIV testing [14]. A total of 67 countries already have policies about partner notification; it could be a valid way to help people identify their risk of HIV infection and promote HIV testing [14]. However, currently, the practicability of partner notification is limited to some extent. The existing partner notification methods, including traditional methods (ie, telephone or address contact), e-postcards sent by email as proposed by Deb Levine et al [15], and website-based partner notification methods [16,17], are aimed at giving HIV-positive individuals an opportunity to tell their sexual partners about their HIV status anonymously after sexual behaviors. As this has been the only type of notification method, partners of HIV-positive people have remained at high risk. In fact, implementing partner notification before sexual behaviors occur might be a fundamental way to avoid HIV transmission between two casual sexual partners and could be defined as primary prevention. However, this strategy has not yet been accepted in practice; one reason is that as a type of healthy behavior, implementing partner notifications is a long and difficult process, but most studies focus on short-term effects [18-20]. In addition, the lag and inconvenience of information exchange of existing partner notification methods was also a defect that needed to be improved [17].

Benefiting from the rapid development and popularization of the Internet, especially the rapid increase of health-related mobile phone apps, there are opportunities and techniques to achieve the goal of partner notification in advance of sexual behaviors [21,22]. In this study, we aim to design and develop a welcoming and convenient mHealth app with a main function of partner notification. We believe that this app could reduce the risk of HIV transmission among MSM. An observational study among app users was implemented to evaluate the effect of the app’s functions.

## Methods

### mHealth App Design and Development Procedures

To meet the goal of promoting partner notification of HIV among MSM, an mHealth app was designed and developed. The app that we sought to develop had to meet the following functions and requirements:

1. Provide a feasible, easy, convenient technical solution to achieve the function of partner notification for MSM before they have sex.
2. Obtain and store users’ HIV statuses and test dates, which can be used for partner notification in health centers by promoting HIV testing; HIV statuses can be approved and updated in a timely manner by health centers.
3. Allow users to query their HIV test results (ie, HIV status) conveniently and in a timely manner.
4. Protect users’ privacy information in an effective, ethical, and nonobtrusive way.
5. Bring minimum economic and psychological burden, as well as a good user experience, to the users.

An interdisciplinary research and development team was formed to design and develop the app, including the following experts: HIV/AIDS epidemiologists, medical workers (ie, doctors and nurses), social workers, and software systems engineers. Team members had repeatedly discussed the implementation of the system functions and determined the final design scheme. The app was primarily developed in Java. The Web app technology was used to integrate some webpages, which combined the advantages of better human-computer interaction with cost-effectiveness in cross-platform development. These webpages were programmed and developed in HTML5.

**Figure 1:**
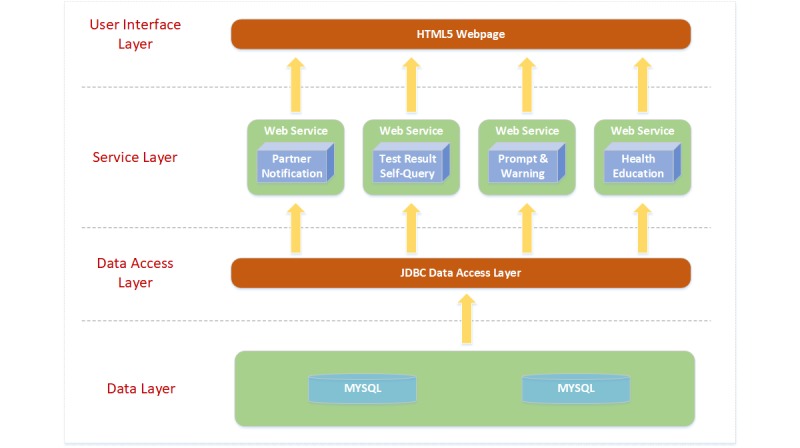
Application architecture. JDBC: Java Database Connectivity; SQL: Structured Query Language.

To improve robustness of the app system, we designed and strengthened the app’s infrastructure, architecture, and processing as follows. First, regarding the infrastructure, we set up system server hosts within the organization that provided HIV testing services. The operating system was Windows Server 2012. Second, we formed a four-layer architecture, including a data layer; a data access layer; a service layer, including four function modules; and a user interface layer (see Figure 1). Third, regarding processing, a log monitor module was set up within the app system to record any invalid inputs and queries by app users, such as incorrect phone number, which could be used to optimize the system. To protect our data, we applied Hypertext Transfer Protocol Secure (HTTPS) communication over our system. The open-source Structured Query Language database MySQL was used for Web data storage, and the Message-Digest algorithm 5 (MD5) encryption technology was applied to encrypt the sensitive data in the database.

Two versions of the app for mobile phones running on iOS and on the Android operating system were developed; the functional webpages could also be accessed by Web browsers. After the completion of app development, we conducted internal and external testing; experts and staff on the project team tested and modified the system repeatedly to develop the final version of app to be used online. Engineers and testers, other than team members, were also invited to review code and test the app’s function modules.

### Observational Study Implementation

An observational study based on the app users’ usage data was carried out to identify the connection among MSM and to evaluate the effectiveness of the app in reducing the risk of HIV infection. The study was implemented in Harbin, Heilongjiang province, China. Participants were enrolled into the study by the KangTong clinic for a period of 1 year between July 1, 2017, and June 30, 2018; the KangTong clinic is a community-based organization for MSM in Harbin and was the health center where app data were stored. The KangTong clinic also provided HIV testing services for the study; the clinic is the largest organization in Harbin that can provide testing services for MSM, except for hospitals and the local Center for Disease Control and Prevention (CDC).

The inclusion criteria for participants were as follows: (1) biologically male, (2) had oral or anal sex with men at least once during their lifetime, (3) 15 years of age or older, (4) had no difficulty using a mobile phone, on which the app must be installed, (5) willing to provide their mobile phone numbers to serve as the unique identification numbers of the app’s self-query and partner notification functions, (6) willing to use the app’s function modules, (7) willing to complete the questionnaire for the study, and (8) willing to complete the informed consent document. MSM who met all the inclusion criteria, but whose first HIV test results were positive, were also invited to register as app users and complete the questionnaire but were not included in the statistical analysis of HIV incidence.

When recruiting participants, the researchers and trained staff from the KangTong clinic publicized the app through online platforms (eg, WeChat, QQ, and Weibo) and offline venues (eg, the clinic, parks, and bars). The app’s modules and their functions were introduced to all of the participants by trained staff. The participants were encouraged to use the app but were not obligated to do so. An electronic informed consent form was provided on the app when participants began to register. After completing the user registration, no specific instructions that would persuade them to use any particular module of the app were given to the participants; however, we observed them using the app in their own way to meet their needs. Participants were also invited to receive HIV testing provided by the KangTong clinic. When they made use of the provided HIV testing services, they needed to fill out a questionnaire containing basic demographic information and recent high-risk sexual behaviors, which they were informed of in the above electronic informed consent form. After completing an HIV test, the results and date were approved and input into the system by the clinic staff.

In order to protect participants’ privacy and confidentiality, the recruitment process, HIV testing services, and participant management after recruitment were all implemented one-to-one by the KangTong clinic’s trained staff, and participants’ real names were not collected during these processes. The HIV test results were communicated to the participants in two ways: first, by communicating the results to participants in person by the clinic’s staff and, second, by allowing participants to query the results themselves through the app’s function module. Though participants gave their phone numbers when they registered as app users, phone numbers would not be shown to clinic staff and staff would not call participants, in order to avoid disturbing them. The geographical locations collected by the app were also not shown to clinic staff.

### Statistical Analysis

In this study, the app users who had at least two HIV test results during the 1-year period, and whose first HIV test results were negative after becoming registered users of the app, were included in the final analysis. The frequency and use of the app’s different modules among these users were summarized. Because the frequency was not a normal distribution, medians and IQRs were calculated to describe the central tendency. The Mann-Whitney *U* test was used to test the difference in median frequencies between the two groups. The chi-square test was used to compare the basic characteristics among the MSM with the usage of different modules.

Several social networks were formed by users with at least two HIV test results and based on their connection with other MSM who were covered by the app’s partner notification system. MSM who requested others’ HIV statuses or whose statuses were requested by other MSM were the nodes of the networks identified by the transformed unique IDs. The directed edges in networks were built according to the request relationships. The nodes were weighted by the number of MSM they connected with, and the edges were weighted by the number of requests between two MSM in the same direction. Their HIV statuses (ie, HIV negative, HIV positive, and unknown status) were also distinguished in the network construction. In addition, HIV seroconversions were marked specifically in networks. We assumed that two MSM who were connected in the networks may be potential sexual partners.

The cumulative HIV incidence of the users with at least two HIV test results was calculated by dividing the total number of seroconversions by the total follow-up periods (ie, the period of time between one participant’s first HIV test to his last HIV test, indicated by person-year). The HIV incidence of these users who used the app for 5 months or less or for more than 5 months was also calculated, of which 5 months was the median time of follow-up shown in the analysis of the study. Poisson regression was used to estimate the risk ratios (reported with 95% CIs and *P* values) of different follow-up periods and different modes of app usage. Because the enrollment time of each participant was different, the follow-up period for each participant was calculated individually. The date of HIV seroconversion was defined as the midway point between the last negative test date and the first positive test date. The users who used the app for more than 5 months were also included in the calculation for HIV incidence of those who used the app for 5 months or less; their follow-up periods were defined as 5 months (0.42 person-years). Regarding the calculation for HIV incidence of users who used the app for more than 5 months, their follow-up periods were the period of time between their first HIV test and their last HIV test.

A two-sided *P* value of .05 or less was regarded as significant. The data from the questionnaires, the self-queries, and the partner notification records that were linked by unique ID were double-checked in Microsoft Access, version 2013, and SPSS, version 21.0 (IBM Corp). Statistical analyses were done with SPSS, version 21.0 (IBM Corp). Network visualization was done with Cytoscape, version 3.5.1 (Cytoscape Consortium). Mapping was done with ArcGIS, version 10.0 (Esri).

### Ethical Issues

This study was approved by the Peking University Institutional Review Board (IRB00001052-16016).

## Results

### App Development: Modules and Functions

The mHealth app named *Golden ark* was developed and contains four main modules: the *partner notification* module, the *test results self-query* module, the *prompt and warning* module, and the *health education* module.

#### Partner Notification Module

**Figure 2:**
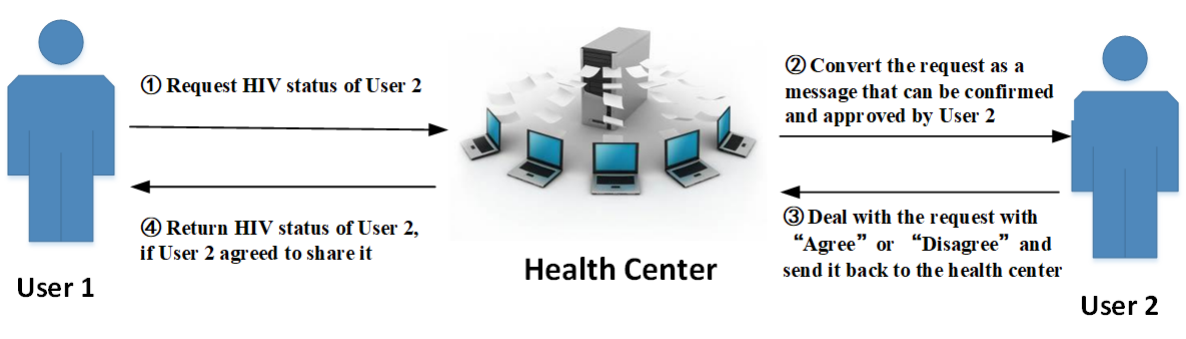
The framework of the partner notification system.

**Figure 3:**
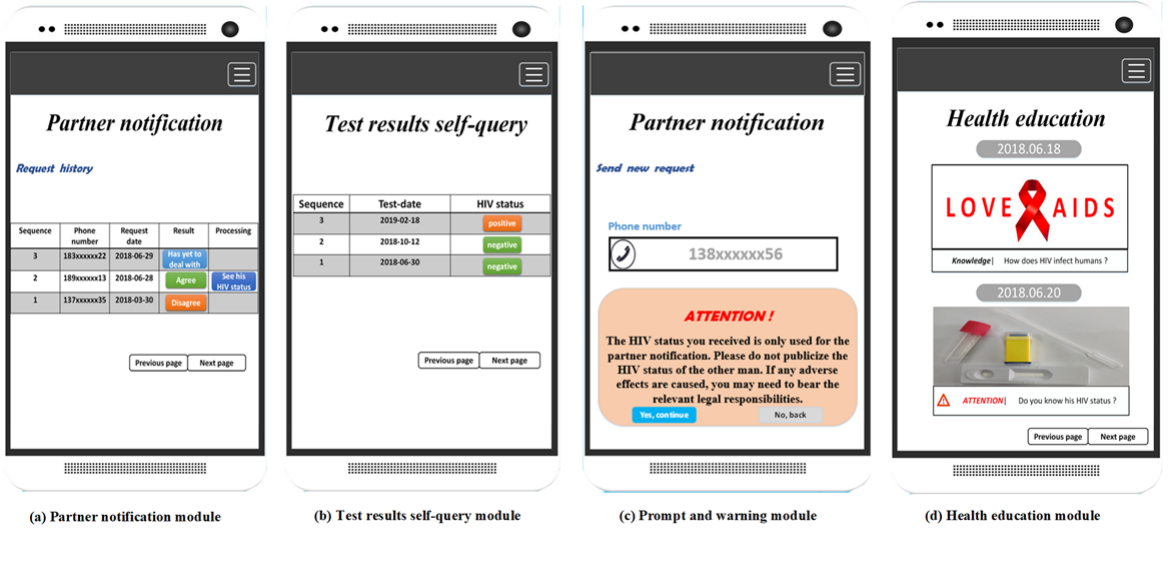
The interfaces of the app’s four modules.

The app provides a function that allows users to request each other’s HIV status from the health center. Figure 2 represents the framework of the partner notification system, which connects the app platform with the health center. The implementation of a request is divided into four steps:

1. User 1 sends a message to the health center requesting User 2’s HIV status through the system; this is a very simple step where User 1 inputs User 2’s phone number into the app’s *Send new request* interface and then submits it.
2. After receiving the request, the health center converts it into a message that can be confirmed and approved by User 2 and sends the message to User 2’s *Receive request* interface.
3. If User 2 agrees to show his HIV status to User 1, he must click on the *Agree* button on his *Receive request* interface within 24 hours; his status will then be sent to User 1. This is deemed a successful request. If User 2 clicks the *Disagree* button or refuses to deal with the request message within 24 hours, the request will be terminated and deemed an unsuccessful request.
4. User 1 can see the real-time processing results through his *Request history* interface as soon as User 2 has finished processing the request, which could have three outcomes: *Agree*, where User 2 agreed to share his recent HIV status; *Disagree*, where User 2 chose not to share his HIV status; and *Has yet to deal with*, where User 2 has not yet dealt with the request. The module’s function can be implemented through several interfaces (see Figure 3, a, and Multimedia Appendix 1).

After the four steps, the two users’ phone numbers, HIV status, the request status (ie, successful or not), and date are recorded by the health center and can be linked to the two users’ questionnaire information by their phone numbers.

We expect that when two MSM are deciding whether to meet, this friendly and convenient communication about HIV status via a social media app will help them decide, in a timely manner, whether to meet and have sex or not. If they use the function of partner notification and find out that one of them is HIV positive or HIV unknown, their meeting may be cancelled. In addition, if one user cannot get the other’s HIV status or the other man has not had an HIV test for over 3 months, he is also more likely to cancel their meeting, thereby reducing his risk for HIV infection.

#### Test Results Self-Query Module

After completing the HIV test in the clinic, users can query their HIV test results through the app's *test results self-query* module from the health center—where all users’ HIV test results are stored—just by clicking the module button on the interface. The test results that can be queried are approved by the health center and will be kept in the system; users can also look up their previous HIV test dates and results. For the convenience of users’ queries, the records are arranged in reverse order according to the HIV test dates: the latest test record is listed at the top (see Figure 3, b). After every self-query, the user’s phone number, HIV status, and self-query date are recorded by the health center and can be linked to the user’s questionnaire information by their phone number. If the test result is positive, users can contact the health center for a follow-up consultation and help.

#### Prompt and Warning Module

On the basis of the self-query and partner notification module, a module aiming to give users some necessary prompts and warnings are embedded into the main functional module to alert users. This module contains two major parts: *dynamic prompt and warning* and *fixed prompt and warning*.

##### Dynamic Prompt and Warning

If one user’s last HIV test result was HIV negative but he does not have a new result stored at the health center within the last 3 months—a 3-month interval is recommended by CDC [13]—the user will receive a prompt to persuade him to come to the health center for HIV testing (see Multimedia Appendix 2, a)

When one user (ie, User 1) requests to view the other user’s (ie, User 2) HIV status, there are five request results possible:

1. User 2 refuses to show his HIV status to User 1: a *Disagree* or *Has yet to deal with* request result.
2. User 2 agrees to show his HIV status to User 1, but User 2 does not have any HIV test results stored in the health center’s system: an unknown HIV status.
3. User 2 agrees to share his status, but User 2 is HIV positive.
4. User 2 agrees to share his status and the recent test result is HIV negative, however, the recorded result was 3 months ago.
5. User 2 agrees to share his status and has an HIV-negative test result recorded within the last 3 months.

According to the five possible request results, the system will give corresponding warning messages. For the first three results, the app module will send a warning message to User 1 to alert him to think carefully about dating User 2 and to pay attention to the potential risks (see Multimedia Appendix 2, b and c; the figures show the third situation as an example). For the fourth result, the module will alert User 1 that User 2’s test result is overdue and he needs to be retested for HIV (see Multimedia Appendix 2, b and d). For the last result, the system will send a warning message to User 1 that alerts him to the possible window period and new infection of User 2 and advises him to use condoms to prevent potential risks of HIV and other sexually transmitted diseases (see Multimedia Appendix 2, b and e).

The above prompts and warnings are shown on User 1’s *Partner’s HIV status* interface when he uses the partner notification module.

##### Fixed Prompt and Warning

Two kinds of fixed prompts and warnings are set up on the app’s interface to remind users to be more cautious and to pay attention to the security of their information. First of all, when one user uses the partner notification module and prepares to submit the mobile phone number of the man for whom he wants to request HIV status, a prompt will appear on the interface to warn him not to publicize the other man’s HIV status (see Figure 3, c). In addition, when one user receives a request message on the *Receive request* interface, if he wants to click on the *Agree* button, he will receive a warning saying “If you click the ‘Agree’ button, your HIV status will be known by the other man; please be cautious” (see Multimedia Appendix 3).

#### Health Education Module

A health education module is provided to give users some necessary and timely health information to prevent them from engaging in high-risk behaviors (see Figure 3, d) This module also contains two parts: *Regular Intervention Information Dissemination* and *Dynamic Intervention Information Dissemination*.

##### Regular Intervention Information Dissemination

Health information will be disseminated to all users in the app regularly (ie, every week) to give users guidance on HIV prevention, such as basic knowledge about HIV/AIDS, skills in HIV/AIDS prevention, policies and regulations for AIDS patients, what and where services can be accessed, and so on.

##### Dynamic Intervention Information Dissemination

Based on the user’s request behaviors (ie, frequency, successful or not, and so on) and his HIV status, as well as the HIV status of the men to whom he sent requests, the system may determine that the user is in a high-risk state. If so, the system will send reminders to the user, in a timely manner on the app’s interface, about paying attention to his security regarding sexual behaviors, the correct method of condom use, the use of the HIV self-testing kit, and nearby HIV testing institutions.

### App Data Collection and Management

The app users’ data are collected from three sources:

1. The regular questionnaires users fill in before their application for HIV testing services, which are stored in the database automatically after they submit them through the app.
2. The approved HIV status and testing date for each HIV test, which are input into the system by the health center’s trained staff and stored in the database for the app’s partner notification module: users cannot input the test results themselves.
3. The self-query behaviors (ie, self-query records), the interactive behaviors (ie, partner notification records) of users, and the geographical locations of the usage of the two modules are automatically stored in the database when these modules are used.

The three kinds of data are linked by users’ phone numbers that were used for registration, which became their unique IDs.

All of the data are stored in an encrypted database by the health center’s stand-alone server in case of information leakage and illegal use. The reason we use phone numbers as unique IDs is that phone numbers are convenient and easy for users to remember as their unique usernames for registration and log-in; this is very common for most apps in China for managing users. Because users’ phone numbers are private information, we encrypt this field when we store it in the database. When we export the data for analysis, this field will be replaced by a 16-bit string made up of random letters and numbers as the transformed unique IDs.

The kinds of information we collect, how we use and protect the information, as well as the possible risks are written in the informed consent form. All users need to sign the electronic informed consent form when they register.

### Observational Study

From July 1, 2017, to June 30, 2018, 3186 MSM were recorded in the health center’s self-query records and/or partner notification records. Of the 3186 MSM, there were 678 users (21.28%) with at least two HIV test results since becoming app users. There were 20 seroconversions found among these 678 users (2.9%).

**Figure 4:**
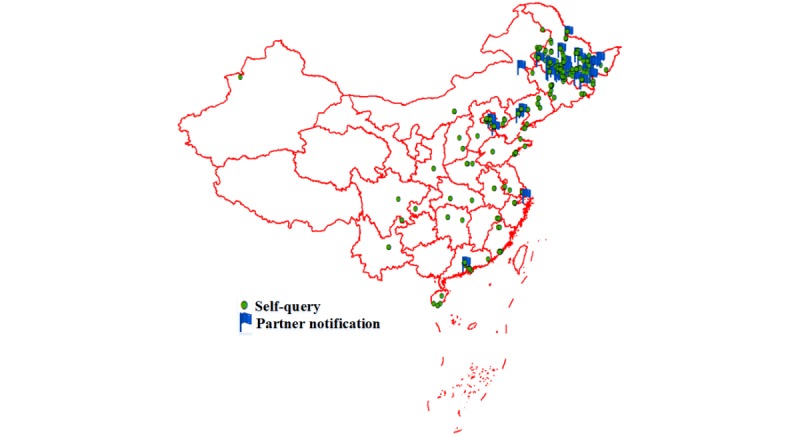
Location distribution of the self-queries and partner notifications.

Of the 678 users, a total of 6473 self-queries (median 6, IQR 2-12) and 623 partner notifications (median 2, IQR 1-3) were recorded. MSM who had used the partner notification function were likely to have more self-queries than those who did not (*P*<.001) (see Table 1). Most (4740/7096, 66.80%) of the self-queries and partner notifications occurred in Harbin, Heilongjiang province, where the health center was located. Over the 1-year study period, usage location of the two function modules covered all of mainland China, except six provinces—Qinghai, Tibet, Gansu, Ningxia, Guizhou, and Guangxi—and was distributed centrally in eastern China (see Figure 4).

**Table 1:** Usage frequency of functions by the app users.

Function		Total	Never used partner notification (self-query only)	Had used partner notification	Mann-Whitney *U*	*P* value
**Self-query**						
	MSM^a^, n (%)	662 (100)	424 (64.0)	238 (36.0)		
	Frequency, n (%)	6473 (100)	3340 (51.60)	3133 (48.40)	35,443	<.001^b^
	Frequency, median (IQR)	6 (2-12)	5 (2-10)	9 (4-16)		
**Partner notification**						
	MSM, n (%)	254 (100)	0 (0)	254 (100)		
	Frequency, n (%)	623 (100)	0 (0)	623 (100)	N/A^c^	N/A
	Frequency, median (IQR)	2 (1-3)	N/A	2 (1-3)		

^a^MSM: men who have sex with men.

^b^Significant at *P*<.05.

^c^N/A: not applicable.

Most of the 678 users were unmarried (536/678, 79.1%), permanent residents (423/678, 62.4%), Han (648/678, 95.6%), living in the local area for more than 2 years (518/678, 76.4%), and had university-level education or above (447/678, 65.9%). Most of the users were homosexual, however, a sizable proportion (130/678, 19.2%) were bisexual MSM. In terms of seeking sexual partners, most of the MSM always used the Internet and some mobile apps to do so (555/678, 81.9%) instead of offline venues, such as gay bars, parks, bathrooms, and so on. The proportions of MSM who were insertive only when having anal sex versus those who had both insertive and receptive roles were similar (273/678, 40.3% vs 250/678, 36.9%) and were both higher than those who were receptive only (155/678, 22.9%). Among the 678 users, 254 (37.5%) of them had used the partner notification function (see Table 2).

**Table 2:** Basic characteristics of the app users.

Characteristics		Total (N=678), n (%)	Never used partner notification (self-query only) (N=424), n (%)	Had used partner notification (N=254), n (%)	Chi-square (df)	*P* value
MSM^a^ (N=678)		678 (100)	424 (62.5)	254 (37.5)		
**Age (years)**					**0.1 (2)^b^**	**.94**
	<25	171 (25.2)	109 (25.7)	62 (24.4)		
	25-40	267 (39.4)	167 (39.4)	100 (39.4)		
	>40	217 (32.0)	139 (32.8)	78 (30.7)		
	Missing data	23 (3.4)	9 (2.1)	14 (5.5)		
**Marital status**					**0.9 (2)^b^**	**.64**
	Married	105 (15.5)	70 (16.5)	35 (13.9)		
	Unmarried	536 (79.1)	331 (78.1)	205 (81.3)		
	Divorced or widowed	32 (4.7)	20 (4.7)	12 (4.8)		
	Missing data	5 (0.7)	3 (0.7)	2 (0.8)		
**Registered residence**					**10.2 (1)**	**.001^c^**
	Permanent residents	423 (62.4)	245 (57.8)	178 (70.1)		
	Migrant	255 (37.6)	179 (42.2)	76 (29.9)		
**Ethnicity**					**0.007 (1)^b^**	**.93**
	Han	648 (95.6)	404 (95.3)	244 (96.1)		
	Minority	26 (3.8)	16 (3.8)	10 (3.9)		
	Missing data	4 (0.6)	4 (0.9)	0 (0)		
**Residence time in local area (years)**					**6.8 (1)**	**.009^c^**
	≤2	160 (23.6)	114 (26.9)	46 (18.1)		
	>2	518 (76.4)	310 (73.1)	208 (81.9)		
**Education**					**1.1 (2)**	**.58**
	Junior high school or below	92 (13.6)	62 (14.6)	30 (11.8)		
	High school	139 (20.5)	85 (20.0)	54 (21.3)		
	University or college or above	447 (65.9)	277 (65.3)	170 (66.9)		
**Sexual orientation**					**0.9 (3)**	**.83**
	Homosexuality	485 (71.5)	305 (71.9)	180 (70.9)		
	Heterosexuality	19 (2.8)	10 (2.4)	9 (3.5)		
	Bisexuality	130 (19.2)	82 (19.3)	48 (18.9)		
	Uncertain	44 (6.5)	27 (6.4)	17 (6.7)		
**Place for seeking sexual partners**					**10.7 (1)**	**.001^c^**
	Internet or software app	555 (81.9)	363 (85.6)	192 (75.6)		
	Offline venues (eg, bars and parks)	123 (18.1)	61 (14.4)	62 (24.4)		
**Sex role**					**6.2 (2)**	**.045^c^**
	Insertive only	273 (40.3)	162 (38.2)	111 (43.7)		
	Receptive only	155 (22.9)	110 (25.9)	45 (17.7)		
	Both	250 (36.9)	152 (35.8)	98 (38.6)		

^a^MSM: men who have sex with men.

^b^Missing data were not included in the test.

^c^Significant at *P*<.05.

Compared with the MSM who only used the self-query function, MSM who had used the partner notification function were more likely to be permanent residents (178/254, 70.1% vs 245/424, 57.8%, *P*=.001), be living in the local area for more than 2 years (208/254, 81.9% vs 310/424, 73.1%, *P*=.009), and take on the insertive role when having anal sex (111/254, 43.7% vs 162/424, 38.2%, *P*=.045). Though the Internet and mobile apps were the main ways for seeking sexual partners, the proportion of MSM in the partner notification group who sought sexual partners in some offline venues was higher than that of the self-query-only group (62/254, 24.4% vs 61/424, 14.4%, *P*=.001) (see Table 2).

**Figure 5:**
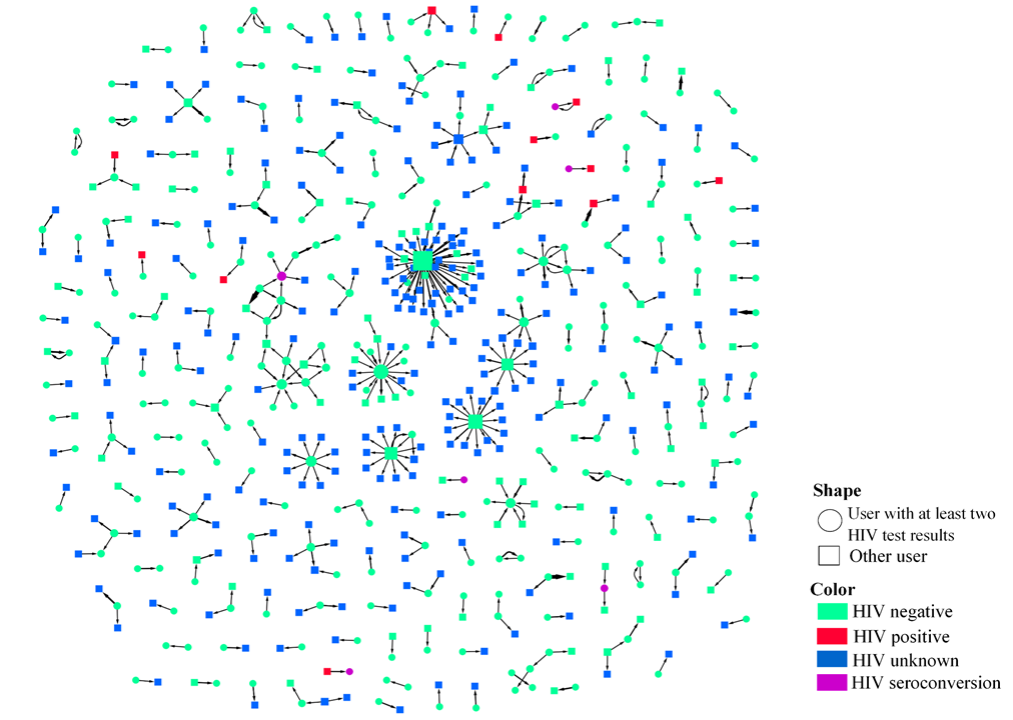
Networks formed by the app’s partner notification module.

**Figure 6:**
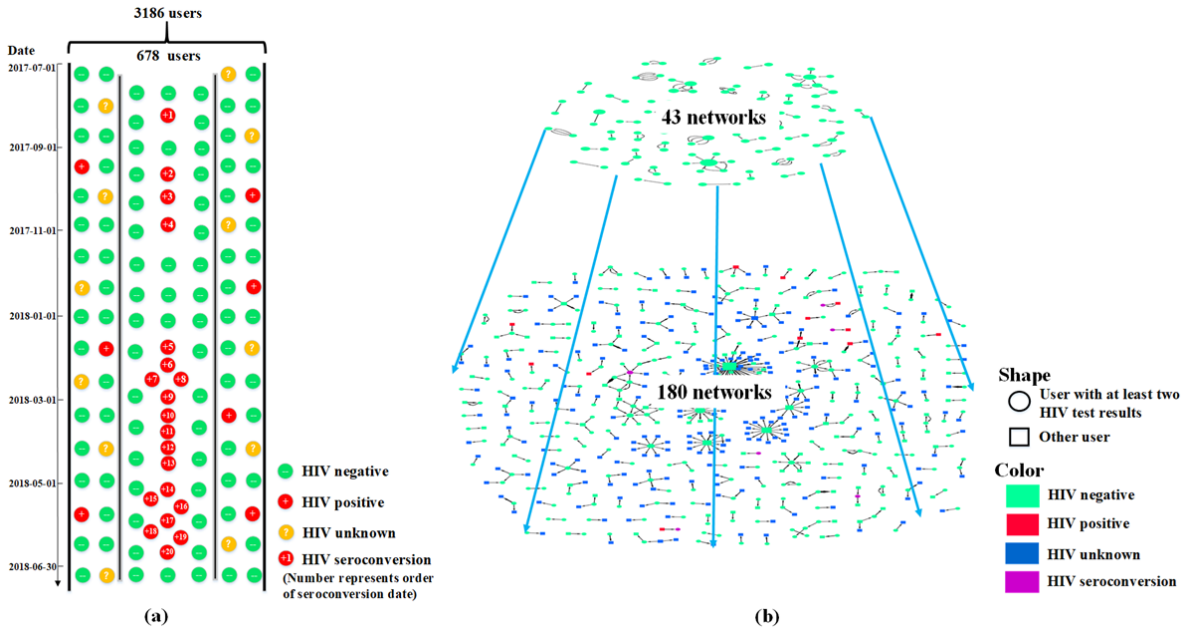
Extension of men who have sex with men (MSM) users and the two-tier networks.

Overall, 180 networks were constructed among 254 users with at least two HIV test results (254/678, 37.5%) and other users they connected with (see Figure 5). Though most of the networks had just two nodes, there were still several larger networks; the common feature of these networks was that they all had one user with a high degree of centrality (ie, number of edges connecting a node to other nodes in the network) requesting the HIV status of other MSM. Some HIV-negative MSM frequently requested the HIV status of other MSM; this occurred a maximum of 55 times with 47 MSM involved. The MSM with a high degree of centrality from almost all of the larger networks were HIV negative. In addition, by using the app and forming networks, we are able to not only achieve our purpose of carrying out research on the 678 target users, but we can also find the relationships among more MSM in addition to the target MSM (see Figure 6, a). If we only focus on the connections among the 678 MSM, there were only 43 networks formed by 109 users with at least two HIV test results (109/678, 16.1%). However, based on the actual situation recorded by the app, this could extend to 180 networks with 254 users with at least two HIV test results and they could find more MSM that connect with them (see Figure 6, b).

**Figure 7:**
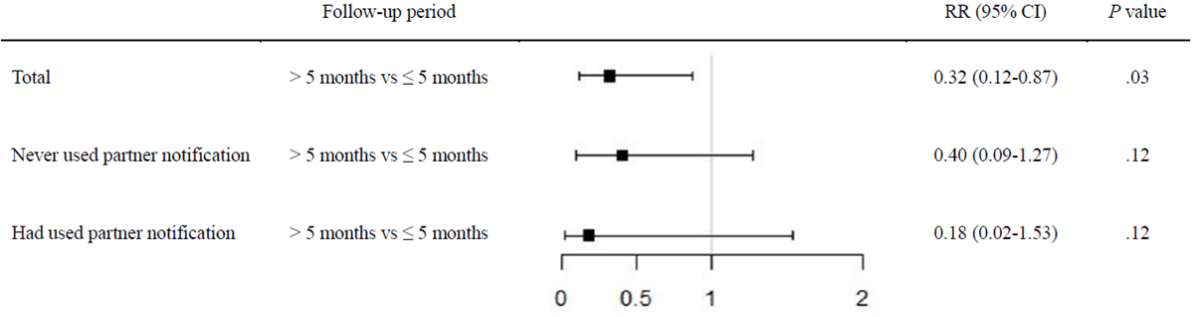
HIV infection risk between different periods of follow-up. RR: risk ratio.

The median follow-up period for the 678 MSM was 5.05 months (IQR 2.82-7.64). The cumulative HIV incidence was 6.75 per 100 person-years (95% CI 4.38-10.01). Of the 20 seroconversions, 6 MSM (30%) had used the partner notification module while the other 14 MSM (70%) only used the self-query module. No significant differences in HIV incidence were found between MSM who had not used the partner notification module (ie, who just used the self-query function) and MSM who had used it. The difference in HIV incidence among MSM with a follow-up period of 5 months or less, or more than 5 months, between the two groups was also not significant (see Table 3). However, regardless of whether this module is used, longer usage (ie, >5 months) of the app could reduce HIV incidence compared with a follow-up of 5 months or less (incidence: 2.22 per 100 person-years vs 6.99 per 100 person-years; risk ratio 0.32, 95% CI 0.12-0.87, *P*=.03) (see Figure 7).

**Table 3:** HIV incidence of app users.

Follow-up period		Total (N=678)	Never used partner notification (self-query only) (N=524)		Had used partner notification (N=254)	Risk ratio (95% CI) (used vs never used)	*P* value
**Total**						**0.78 (0.27-2.29)**	**.65**
	MSM^a^ (N=678), n (%)	678 (100)		524 (77.3)	254 (37.5)		
	Seroconversion, n (%)	20 (2.9)		14 (2.7)	6 (2.4)		
	Person-years	296.47		177.53	118.94		
	Incidence per 100 person-years (95% CI)	6.75 (4.38-10.01)		7.89 (4.73-12.52)	5.04 (2.37-9.81)		
≤**5 months**						**0.52 (0.06-4.68)**	**.56**
	MSM (N=678), n (%)	678 (100)		524 (77.3)	254 (37.5)		
	Seroconversion, n (%)	15 (2.2)		10 (1.9)	5 (2.0)		
	Person-years	214.48		130.83	83.65		
	Incidence per 100 person-years (95% CI)	6.99 (4.26-10.95)		7.64 (4.20-13.06)	5.98 (2.63-12.24)		
**>5 months**						**0.64 (0.25-1.66)**	**.36**
	MSM (N=678), n (%)	348 (51.3)		283 (41.7)	142 (20.9)		
	Seroconversion, n (%)	5 (1.4)		4 (1.4)	1 (0.7)		
	Person-years	225.01		131.36	93.65		
	Incidence per 100 person-years (95% CI)	2.22 (0.98-4.55)		3.05 (1.24-6.67)	1.07 (0.26-3.94)		

^a^MSM: men who have sex with men.

## Discussion

### Principal Findings

The mHealth app with four modules seemed to be favored by MSM. Over a 1-year project duration, the app reached 3186 MSM, of which 678 uploaded at least two HIV test results and 37.5% used the partner notification function. The *partner notification* module provided a system connecting MSM and the health center, which could enable the function of sharing HIV statuses between potential sexual partners, after permission was given, in a way that preserved privacy. Such a module can enable partner notification before two MSM meet and have sex, and it can minimize embarrassment and anxiety after meeting. It is well aligned with the WHO’s guidelines for sensitivity and confidentiality [23]. The *test results self-query* module enabled MSM to get the approved test results in time and to seek further guidance and intervention. In addition, the approved results provided a trustworthy source of users’ HIV statuses when they used the *partner notification* function, which was also aligned with the *Know Your Status* theme of World AIDS Day 2018 [24]. The *prompt and warning* module and the *health education* module are two important auxiliary modules that can protect users from high-risk behaviors and teach users more skills in HIV/AIDS prevention. Taking advantage of the rapid development of the Internet and health-related mobile apps, this app and the app-based intervention have been developed to be much more convenient, user friendly, and cost-effective than traditional methods, and is easier to implement; the app is an expansion of the traditional behavior intervention mode, which focuses on education [25,26].

Our study has explored a new method of partner notification that could be implemented before high-risk sexual behaviors occur. In 2016, the WHO’s evidence-based guidelines recommended assisted HIV partner notification services. This refers to the assistance of consenting HIV-positive clients by a trained provider to disclose their status or to anonymously notify their sexual partners of their potential exposure to HIV infection and offer voluntary HIV testing services. These notification services could increase HIV testing among partners of HIV-positive people and result in a high proportion of HIV-positive people being diagnosed and linked to care [14,27-30]. However, some limitations might challenge the effectiveness of assisted partner notification: first, it attaches importance to the notification after sexual behavior has occurred, which might not prevent HIV transmission to the partners of HIV-positive individuals; second, assisted partner notification emphasizes the participation of health workers, which requires more time, human, and economic resources, and HIV-positive individuals might not be willing to cooperate [31,32]. Our method of partner notification could overcome these limitations to some extent. As well, two auxiliary modules of the app might intensify the effect of partner notification and MSM’s sense of healthy self-responsibility, which could facilitate the transition from secondary prevention to primary prevention of HIV transmission among MSM.

Based upon connections and communications among MSM, the social networks were formed and analyzed to show the true epidemiologic behaviors among MSM. The networks formed by the partner notification connections could help find more MSM and their connections, which are more real, objective, and comprehensive than the connections found by traditional observational or cohort studies. Some much bigger networks were marked and the MSM with a high degree of centrality were more active than the MSM they were connected with. Though the MSM with a high degree of centrality were almost all HIV negative in our study, their active behaviors might put them at high risk of HIV infection; if they turned out to be HIV positive, active sources of infection would emerge. Thus, the MSM with a high degree of centrality in the network should be given a targeted intervention. The network was not only the reflection of the MSM’s connection, as peer pressure and peer norms might also exist in social networks; several studies have shown that relying on the connections and communications among the members of the networks could promote behavior change and reduce the risk of HIV transmission [33-35]. Therefore, the networks formed by true epidemiologic data could be used to detect individual risk behaviors to make targeted policies for HIV control among MSM.

In this study, we were glad to find that the risk of HIV infection was decreasing. The cumulative HIV incidence was 6.75 per 100 person-years (95% CI 4.38-10.01), which was slightly lower than 7.1 per 100 person-years —the incidence of a cohort study with an intensive preventive intervention for MSM in Beijing, China [36]. The longer the app was used, the lower the risk of seroconversion. More than 5 months of app usage could reduce the HIV incidence to a much lower level of 2.22 per 100 person-years (95% CI 0.98-4.55), which was lower than the reported incidence in most regions of China and showed the potential effect of the app in reducing MSM’s HIV infection risk [37]. In addition, the users of the app may naturally expand the intervention to more MSM than expected. In our study, the 254 users with at least two HIV test results connected with more MSM with no or only one HIV test result. The connections could be a process of expanding the reach of the intervention; it would encourage the other MSM to adopt this mode and to get regular HIV testing, which may increase the effect.

### Limitations

Though the app has good application values, several limitations exist. We did not apply the intervention of HIV testing intervals to users, so some users’ HIV testing intervals were too long to truly reflect their current HIV status. For some MSM with high-frequency sexual behaviors, the 3-month interval set in the app’s prompt and warning module may be a bit longer. A dynamic adjusted prompt and warning interval based on the user’s social network and HIV testing frequency is needed in the module. Another limitation was that, to some extent, the follow-up period of the study was short so there may exist a bias leading to misestimation of the HIV incidence. In addition, the only health center in this study was limited in its capability to provide enough services and spread the app to a much larger MSM population in a short time, which was also less convenient for MSM living in different cities.

### Future Research Prospects

In future research, the following improvements will be made:

1. Improving the app functions to give more specific prompts and health education and to set some limits in search and match frequencies for app users with positive and unknown HIV statuses.
2. A new HIV testing model will be introduced to the health center to make the testing more convenient. In that model, app users will make an appointment to receive the HIV rapid test reagent kit from the health center, which can be sent to their home. After self-testing, users need to take photos of the test reagent kit and upload them through the app interface. The health center staff will review the photos and input the HIV status and date into the app system, which can then be used for test result self-query and partner notification.
3. More sexually transmitted diseases, such as syphilis, hepatitis B, and hepatitis C, will be included in testing services, the self-query module, and the partner notification module to ensure maximum protection levels for MSM.
4. More health centers in additional cities will be included in future studies to improve the convenience of testing services. We will recruit a larger number of MSM participants and increase the follow-up period to verify the effect of the intervention via the app. A consolidated database will be set up to collect and summarize the data from different health centers and to make the self-query and partner notification functions possible across health centers and cities.
5. We will try to cooperate with some popular MSM dating geosocial networking apps to attract more users and expand the population that our intervention can reach.

### Conclusions

To our knowledge, our study is the first of its kind to provide an app to achieve mutual querying of HIV status between two MSM before sexual behaviors, with the integration of HIV testing, partner notification, health center services, and social networks. We are optimistic that our app will be accepted by MSM users and that it will promote partner notification and reduce the risk of HIV infection. This app has the potential to help health care policy makers make targeted policies for HIV control among MSM.

## Appendix

Multimedia Appendix 1 The other interfaces of the partner notification module.

Multimedia Appendix 2 The interfaces of the dynamic prompt and warning module.

Multimedia Appendix 3 The other interfaces of the fixed prompt and warning module.

## Abbreviations

CDC: Center for Disease Control and Prevention
HTTPS: Hypertext Transfer Protocol Secure
MD5: Message-Digest algorithm 5
MSM: men who have sex with men
SQL: Structured Query Language
WHO: World Health Organization
