# Supplementary figures and images for: Protecting Men Who Have Sex With Men From HIV Infection With an mHealth App for Partner Notification: Observational Study
Source: JMIR Mhealth Uhealth. 2020 Feb 19;8(2):e14457. doi: 10.2196/14457 (PMC7057823; doi:10.2196/14457)

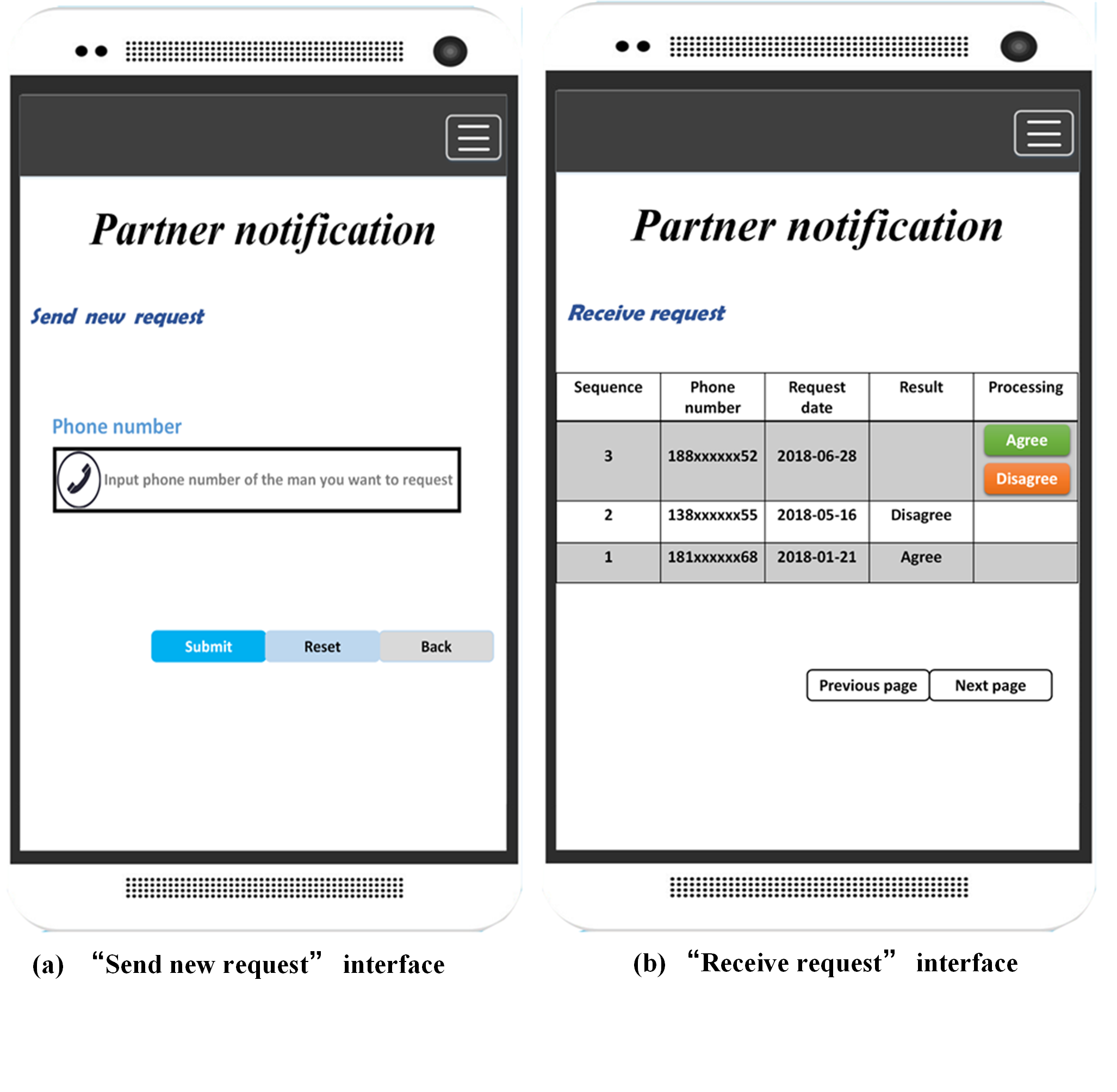

Supplement: Multimedia Appendix 1 [file mhealth_v8i2e14457_app1.png]

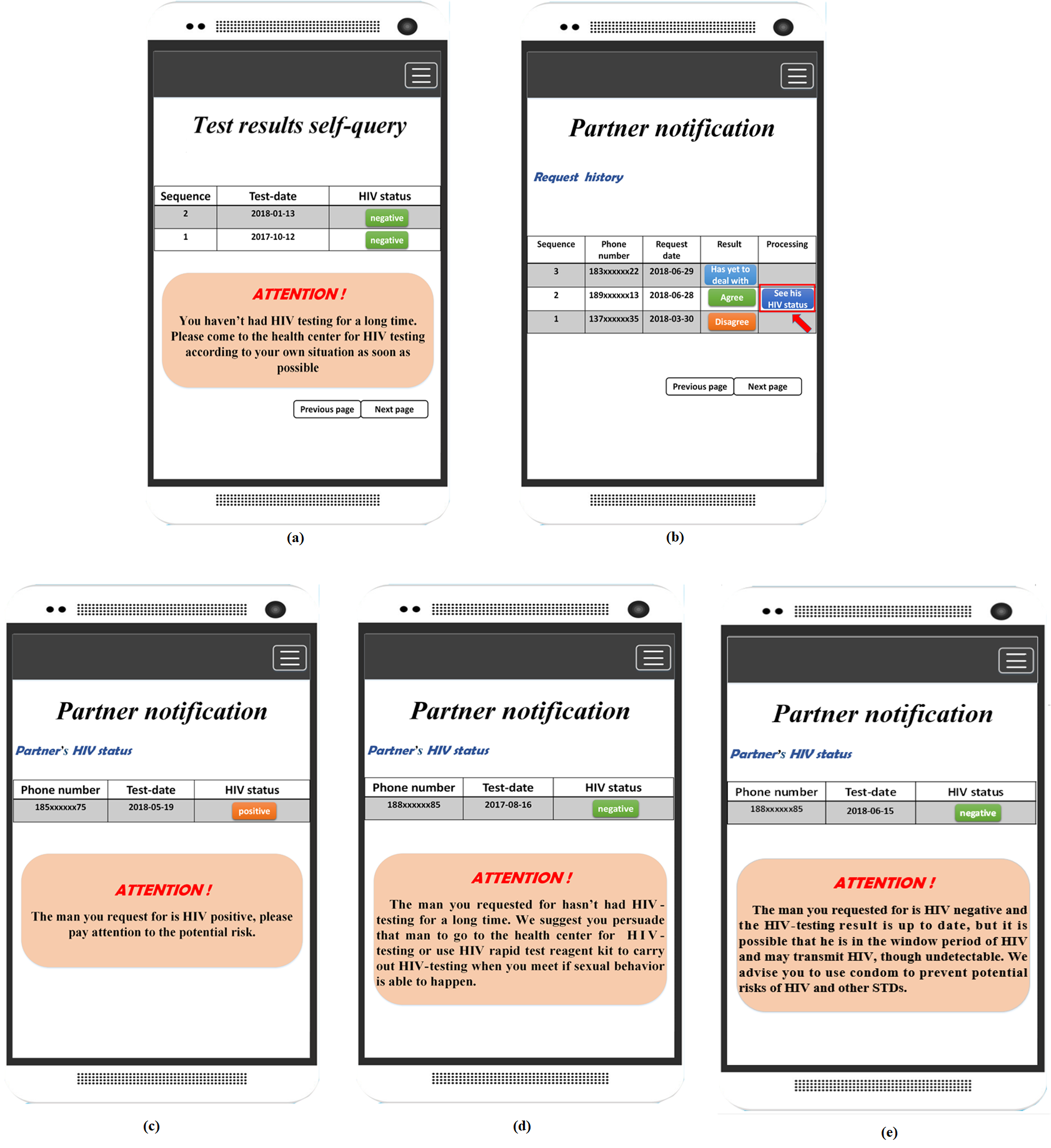

Supplement: Multimedia Appendix 2 [file mhealth_v8i2e14457_app2.png]

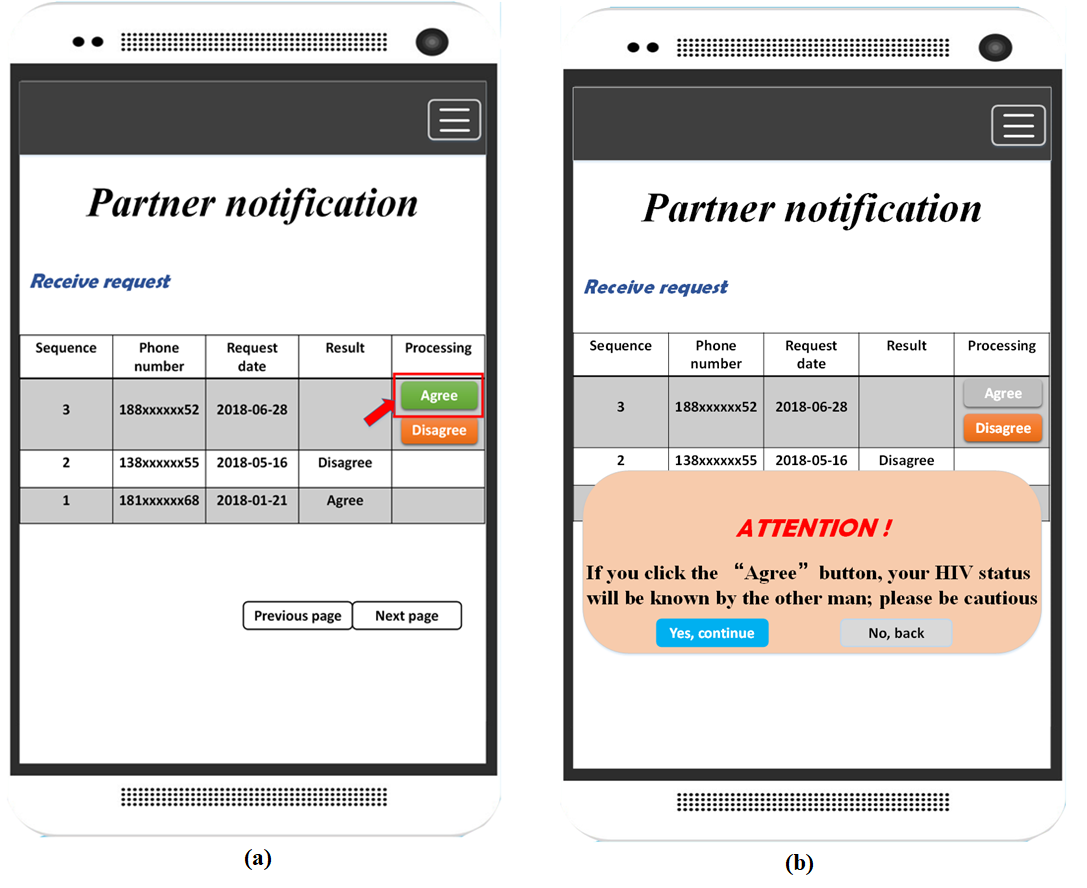

Supplement: Multimedia Appendix 3 [file mhealth_v8i2e14457_app3.png]
